# Ocular surface health and disease: insight from single-cell RNA sequencing

**DOI:** 10.3389/fgene.2025.1683167

**Published:** 2025-11-11

**Authors:** Shangkun Ou, Minqing Cai, Yuchong Feng, Sijie Lin, Xueer Zheng, Su Zhao, Hao Gu, Yiming Wu

**Affiliations:** 1 Department of Ophthalmology, The Affiliated Hospital of Guizhou Medical University, Guiyang, China; 2 Xiamen Eye Center and Eye Institute of Xiamen University, School of Medicine, Xiamen, China; 3 Fujian Provincial Key Laboratory of Ophthalmology and Visual Science, Fujian Engineering and Research Center of Eye Regenerative Medicine, School of Medicine, Xiamen University, Xiamen, Fujian, China; 4 Department of Ophthalmology, The Qinglong County People’s Hospital, Qinglong, Guizhou, China; 5 National Engineering Research Center of Ophthalmology and Optometry, Eye Hospital, Wenzhou Medical University, Wenzhou, China; 6 State Key Laboratory of Ophthalmology, Optometry and Visual Science, Eye Hospital, Wenzhou Medical University, Wenzhou, China; 7 National Clinical Research Center for Ocular Diseases, Eye Hospital, Wenzhou Medical University, Wenzhou, China; 8 Department of Biomedical Sciences, School of Infection, Inflammation and Immunology, College of Medicine and Health, University of Birmingham, Birmingham, United Kingdom

**Keywords:** biomarker, cornea, conjunctiva, Fuchs corneal endothelial dystrophy, lacrimal gland, meibomian gland, ocular surface, single-cell RNA sequencing

## Abstract

Ocular surface tissues, primarily consisting of the cornea, meibomian glands, conjunctiva and lacrimal glands, are crucial components of the eyes and are in direct contact with external environment. Various ocular surface abnormalities can lead to ocular surface diseases, and in severe cases, blindness. The intricate diversity of cell types and states, along with the absence of definitive biomarkers for ocular surface tissues, has posed significant challenges to fully understanding corneal stability, disease mechanisms, and therapeutic development. Single-cell RNA sequencing (scRNA-seq) is an advanced analytical technique used to examine the transcriptomes of individual cells. It enables detailed analysis of complex cellular dynamics, the distinction of various cell types, and the discovery of new biomarkers, thus deepening our insight into diverse cellular behaviors. Currently, scRNA-seq is mainly applied to study the developmental processes of ocular surface cells and to explore the pathogenic mechanisms of related diseases, such as dry eye disease, pterygium, keratoconus, Fuchs corneal endothelial dystrophy, ocular graft-versus-host disease, and primary acquired nasolacrimal duct obstruction, which involve the cornea, conjunctiva, and lacrimal gland. This review summarizes the principles and applications of the scRNA-seq technique, including its mechanism, effects, limitations, and applications in ocular surface research, aiming to bridge the gap between incomplete understanding and rapid technological progress of scRNA-seq.

## 1 Introduction

The ocular surface is formed by continuous epithelial tissues with distinct regional specialization, such as the cornea, meibomian glands, conjunctiva, and lacrimal glands (Cher, 2014; Ou et al., 2022). These components serve as crucial protective barriers for the eye. The cornea, which is transparent and lacks vasculature, plays a vital role in providing both transparency and refraction, thereby enabling precise focusing of light onto retinal photoreceptors (Sridhar, 2018). The conjunctiva, in contrast to the cornea, is a semi-translucent mucous layer lining the inner eyelid and extending across the anterior sclera to the corneal boundary (Weingeist, 1973). Characterized by heightened vascularity, it hosts conjunctival goblet cells, which can synthesize and secrete soluble mucin within the tear film (Swamynathan and Wells, 2020; Dong et al., 2009). This mucin, in turn, supports lubrication and protective functions. The tear film, a thin protective liquid layer atop the ocular surface’s epithelial cells, is composed of several constituents (Pflugfelder and Stern, 2020; Li et al., 2022). It comprises a lipid layer from the meibomian glands, an aqueous layer primarily secreted by the lacrimal glands and partly by the conjunctival epithelium, and a mucin layer originating from conjunctival goblet cells (Swamynathan and Wells, 2020). Beyond their structural and biochemical roles, these ocular surface components function as an integrated unit. Their homeostasis relies on coordinated regulation by neural, vascular, endocrine, and immune pathways, which are essential for preserving corneal clarity and stable vision. When these regulatory mechanisms are disrupted, clinical manifestations often follow, most commonly in the form of dry eye disease or keratitis. Moreover, because ocular surface tissues are directly exposed to the external environment, they remain particularly vulnerable to injury and environmental insults. As a result, the prevalence of ocular surface disorders, including dry eye disease, pterygium, and keratoconus, has been steadily increasing. These conditions not only produce symptoms such as redness, irritation, and dryness, but in severe cases may compromise corneal integrity, impair vision, and even lead to blindness (Craig et al., 2017). Therefore, a comprehensive exploration of the heterogeneity and complexity of different ocular surface cells is imperative.

Single-cell RNA sequencing (scRNA-seq) technology has become a leading powerful tool for uncovering the heterogeneity and complexity of RNA transcripts at the single-cell level (den Braanker et al., 2021; Papalexi and Satija, 2018). It also enables the identification of various cell types and their functions within a single tissue or organ (Borner et al., 2021; Jovic et al., 2022). Since its inception, scRNA-seq research has provided substantial insights across various fields, yielding significant advancements in our understanding of cell composition and function in humans, animals, and plants (Tang et al., 2009). Recently, Ahsanuddin and Wu (2023) conducted an extensive review of single-cell transcriptomics studies focusing on the ocular anterior segment, summarizing datasets, experimental considerations, and clinical relevance. Similarly, Arts et al. (2023) reviewed scRNA-seq applications specifically in corneal biology, highlighting the current understanding of corneal cell states and key biomarker genes. Our review builds upon these prior works by not only summarizing scRNA-seq studies of normal ocular surface tissues, such as the cornea, lacrimal gland and conjunctiva, but also extending the discussion to include a disease-oriented perspective. We critically analyze how scRNA-seq has contributed to uncovering cellular and molecular changes in ocular surface diseases, including dry eye disease, keratoconus, and lacrimal gland dysfunction. Furthermore, we provide an in-depth evaluation of technical limitations and biological challenges specific to ocular surface tissues and propose future directions including integrating spatial transcriptomics and multi-omics approaches for improving the understanding of ocular surface health and disease.

## 2 scRNA-seq technology

### 2.1 The working principle of scRNA-seq technology

Cells are the fundamental units of structure and functionality in organisms. Remarkably, each cell possesses its own distinct identity, even within a homogeneous tissue, potentially expressing a unique set of genes. While traditional methods such as microarray analysis (Yao et al., 2022) and bulk RNA sequencing (Roodnat et al., 2022) have been employed to ascertain the average gene expression levels across all cells, these techniques fall short in discerning the intricate disparities in gene expression at the individual cell level. Consequently, they fail to unveil the underlying complexities of cellular changes. The advent of scRNA-seq technology marks a significant stride in research methodology, offering the capacity to illuminate the diversity of each cell, even though their presence within the same tissue and close spatial proximity (Voigt et al., 2019). By enabling biological investigations at a microscopic level, this technique resolves previously insurmountable queries, such as detecting subtle transcriptional changes within heterogeneous cell populations and uncovering the unique molecular identity of individual cells (Hedlund and Deng, 2018).

The scRNA-seq workflow involves steps such as single-cell isolation, cell lysis, reverse transcription, cDNA amplification, and preparation of sequencing libraries (Kolodziejczyk et al., 2015). Among these steps, the initial one, the isolation and capture of single cells, assumes paramount importance. This process commences with the isolation of individual cells, achieved through enzyme treatment, laser capture microdissection, or patch clamping (Hedlund and Deng, 2018). Single-cell capture is a pivotal endeavor aimed at obtaining cells of exceptional quality. A range of capture techniques are commonly employed, such as fluorescence-activated cell sorting (Polisetti et al., 2022), limiting dilution (Kawakita et al., 2008), microfluidic system (Junker and van Oudenaarden, 2015), magnetic‐activated cell sorting (Du H. et al., 2023), and laser microdissection (Sutherland et al., 2018). Following isolation and capture, reverse transcription converts cellular RNA into first-strand cDNA. It is then subjected to amplification via PCR or *in vitro* transcription, resulting in second-strand synthesis (Kolodziejczyk et al., 2015). After barcoded cDNAs are produced from single cells or single nuclei, they can be sequenced using various sequencing platforms (Kleino et al., 2022; Shi et al., 2023). Subsequently, the sequencing data undergo quality control, normalization, and dimensionality reduction, followed by clustering and differential expression analyses, through which cell subpopulations are identified and disease-related molecular signatures in ocular tissues are revealed.

### 2.2 The importance of scRNA-seq technology

First discovered in 2009 (Tang et al., 2009), scRNA-seq has become an important technology in biomedical and clinical research fields. It now serves as a robust technology for detecting and characterizing novel or rare cell types and subpopulations in tissues and organs from different species. To date, it has provided invaluable insights encompassing developmental processes (Tang et al., 2024), tumorigenesis (Amin et al., 2024), immunology (Xia et al., 2025), microbiology (Jia et al., 2024), and a multitude of other domains within the spectrum of health and disease. Despite its achievements, the clinical research via scRNA-seq remains nascent, centering predominantly on augmenting our comprehension of disease processes and the identification of biomarkers for diagnosis and therapeutic intervention.

## 3 Application of scRNA-seq on the ocular surface

### 3.1 Cornea

Structurally, the cornea is divided into the epithelium, Bowman’s layer, stroma, Descemet’s membrane, and the endothelial cell layer. Due to the development of scRNA-seq, the fate of each layer of corneal cells can now be explored, enabling researchers to further understand their similarities and differences at the molecular level (Arts et al., 2023; Sun et al., 2023). One example is the study by Wu et al. (2023), who performed unbiased clustering of scRNA-seq data from 14,732 cells across 40 corneas, identifying 17 clusters corresponding to six major cell types: nine epithelial, three keratocyte, two corneal endothelial, and one cluster each of immune, vascular endothelial, and fibroblast cells. Swarup et al. (2023) used scRNA-seq to study corneal organoids during development and determined the specific time points at which these different cell types appear in fetal corneal development. They found that certain pluripotent cell clusters are committed to the epithelial cell lineage at 1 month, with corneal epithelial, endothelial, and stromal cell biomarkers being expressed. By 3 months, keratocytes become more specified, and by 4 months, epithelial cells are further differentiated. Building upon these findings, the following sections delve into single-cell RNA sequencing analyses of individual corneal layers.

#### 3.1.1 Corneal epithelium

##### 3.1.1.1 XYZ theory of corneal epithelium

Serving as the eye’s first line of defense, the corneal epithelium protects against external pathogens and environmental exposure. It is regularly replenished throughout life, primarily mediated by corneal epithelial stem cells (Schermer et al., 1986). These cells, also known as limbal stem cells (LSCs), reside at the base of the limbus at the junction of the cornea and conjunctiva and are characterized by slow cell cycle, nucleoplasmic ratio, small size and high proliferative potential (Sun et al., 2023; Long et al., 2024). Through proliferation and differentiation, LSCs generate corneal transient amplifying cells (TACs) and limbal progenitor cells (LPCs). TACs move toward the center and surface of the corneal epithelium, eventually developing into adult corneal epithelial cells (Davanger and Evensen, 1971), as defined by the XYZ theory (Thoft and Friend, 1983). ScRNA-seq technology has been utilized to study various aspects of the corneal epithelium, particularly in elucidating the developmental trajectories within this intricate tissue.

**FIGURE 1:**
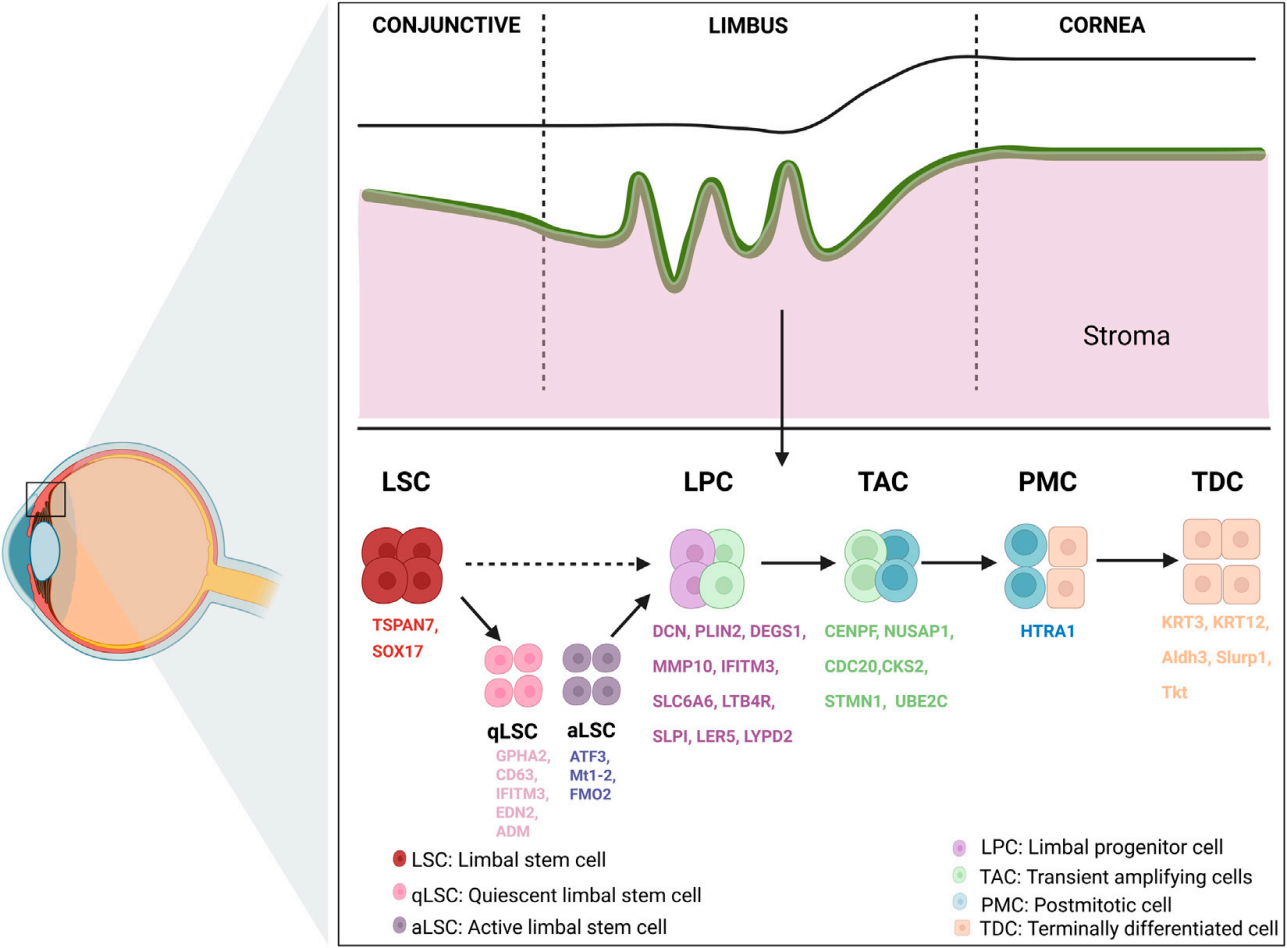
Overview of limbal cornea structure and biomarkers. The limbal region harbors LSCs, which play a central role in maintaining corneal epithelial homeostasis according to the XYZ theory. LSCs, residing in the basal layer of the limbus, give rise to a hierarchical lineage of progeny including qLSCs, aLSCs, LPCs, TACs, PMCs, and TDCs. LSC, limbal stem cell; qLSC, quiescent limbal stem cell; aLSC, active limbal stem cell; LPC, limbal progenitor cell; TAC, transient amplifying cell; PMC, post-mitotic cell; TDC, terminally differentiated cell. Created in BioRender. Wu, Y. (2025).

In 2019, Kaplan et al. (2019) applied scRNA-seq to categorize mouse corneal limbal/corneal epithelial cells into distinct classes: stem/early transient amplifying (TA) cells, mature TA cells, and relatively differentiated cells. This categorization was based on variations in gene expression related to stemness, proliferation, and differentiation. Following this, Li DQ. et al. (2021) extracted 16,360 single cells from the basal limbus of the human cornea for sequencing. Subsequent heterogeneity analysis facilitated the classification of these cells into five primary types: LSCs, LPCs, TACs, postmitotic cells (PMCs, biomarker: *HTRA1*), and terminally differentiated cells (TDCs, biomarkers: *KRT3* and *KRT12*). Subsequently, Altshuler et al. (2021) combined scRNA-seq with quantitative lineage tracing to delineate mouse LSC populations located in the limbal subregion, identifying them as “outer” and “inner” LSCs. The “outer” LSC population contains slow-dividing quiescent LSCs (qLSCs), involved in wound healing and boundary formation, as well as in eco-t-cell regulation. The “inner” LSC population contains actively renewing LSCs (aLSCs), playing an important role in maintaining corneal epithelial homeostasis. Around the same time, Song et al. (2022) mapped the heterogeneous cell populations of corneal limbal cells under normal homeostatic conditions and during the wound healing process. They found that two different types of LSCs might participate in the damage repair process: “putative active LSCs” that actively divide during damage and “putative quiescent LSCs” that remain quiescent. As noted earlier, the recent report by Wu et al. (2023) identified nine epithelial cell subtypes, including qLSCs, TACs, differentiated cells from the cornea, and two minor conjunctival epithelial clusters. These findings are corroborated by other publications mentioned above. These works contribute to refining the XYZ theory of corneal epithelium and establish a foundational understanding of the differentiation trajectory of corneal LSCs (Figure 1).

However, the designation of “LSCs” in many studies warrants careful interpretation. Current evidence primarily relies on gene expression profiles rather than functional validation of self-renewal or multipotency. It remains possible that the so-called LSCs might represent a spectrum of progenitor cells at different stages of activation rather than a distinct stem cell population. This distinction is critical, particularly in the context of limbal stem cell deficiency, where the true absence of a pluripotent stem cell population has not been definitively established.

##### 3.1.1.2 New biomarkers of different developmental stages of corneal epithelial stem cells

Although some biomarkers have been identified for corneal limbal basal cells, distinguishing LSCs from other epithelial cell types continues to be challenging. The advent of scRNA-seq technology has opened new avenues for the accurate identification of LSCs and their diverse developmental stages. Li DQ. et al. (2021) validated the outcomes of human corneal limbal scRNA-seq through RNA scope HiPlex and antibody staining, discovering that SOX17^+^ and TSPAN7^+^ cells were primarily confined to corneal limbal basal epithelium. *TSPAN7* and *SOX17* were identified as novel LSC markers, functionally verified through siRNA-mediated knockdown assays that impaired proliferation and epithelial regeneration. Additionally, LPC-enriched genes were identified, including *SLPI*, *LTB4R*, *SLC6A6*, *IFITM3*, *MMP10*, *DEGS1*, *PLIN2* and *DCN*. In addition, Català et al. (2021) identified possible biomarkers of the human corneal limbal stem cell ecological niche: *CAV1*, *CPVL*, *HOMER3*, *CXCL14*, and genes unique to TACs: *CKS2*, *STMN1*, and *UBE2C*. Two differentiated (*GPHA2* ^+^ and *KRT6B* ^+^) and two highly stem-like (*TP63* ^+^ and *CCL20* ^+^) cell states of corneal limbal stem/progenitor cells were further defined by Dou et al. (2021). In the same year, *CDC20*, *UBE2C*, *NUSAP1* and *CENPF* were discovered for the first time as biomarkers for TACs by Li JM. et al. (2021) using scRNA-seq on 16,360 limbal epithelial basal cells from humans. Further analysis of scRNA-seq also identified potential initiators of these makers changes. Zhu et al. (2023) found the *FEZ1*-*DKK1* axis is crucial in regulating proliferation and senescence of corneal epithelial cells. Sun et al. (2024) found *Creb5*, a transcription factor, is present in LSCs and markedly increases in expression after injury. Recently, Vattulainen et al. (2024) used scRNA-seq to investigate the heterogeneity in the process of differentiating human pluripotent stem cells toward LSCs. Their study identified distinct cell clusters, including a robust induced LSC population expressing important LSC biomarkers such as *TP63* and *KRT14*. Importantly, they proposed *AREG* and *ITGA6* as novel surface biomarkers to enrich for LSC-like cells and improve differentiation consistency across different hPSC lines.

The scRNA-seq results of qLSCs and aLSCs further reveal the changes in makers during the transitional phase. Applying *in situ* hybridization probes to label mouse conjunctival and corneal basal and suprabasal cells, respectively, Altshuler et al. (2021) found that Gpha2 staining could divide the corneal limbus into qLSCs and aLSCs, which were labeled by Gpha2, Cd63, Ifitm3, and Atf3, Mt1-2, respectively. Song et al. (2022) applied scRNA-seq to identify heterogeneous cell populations at the corneal limbus under normal homeostasis and following injury. Their investigation unveiled “putative quiescent LSCs” (biomarkers: *Edn2* and *Adm*) and “putative active LSCs” (biomarkers: *Fmo2*). They also identified diverse LPC subtypes through distinct biomarkers such as *Ler5* and *Lypd2*. Additionally, they noted a progressive increase in the expression of biomarkers associated with mature corneal epithelium (*Aldh3*, *Slurp1*, and *Tkt*) during corneal epithelial maturation.

Nevertheless, the specificity and functional significance of these biomarkers require further investigation. Many of the biomarkers proposed for LSCs and their progeny, such as TSPAN family members and UBE2C, are also associated with general epithelial proliferation in other tissues (Liu et al., 2020; Zhang H. et al., 2024). Therefore, it is essential to distinguish whether these biomarkers define distinct cell populations or merely reflect dynamic cellular states during differentiation or injury response.

##### 3.1.1.3 scRNA-seq for corneal epithelial wound healing

In scRNA-seq studies of the cornea, analyses focusing on corneal epithelial wound healing are common, as this process directly captures the key cellular players involved as well as the differentiation trajectory from LSCs to corneal epithelial cells (Sun et al., 2024; Mirmoeini et al., 2023; Zhou et al., 2024; Yin et al., 2025; Lu et al., 2025). In an earlier study, Mirmoeini et al. (2023) employed scRNA-seq on dissociated healthy, de-epithelialized, and denervated corneal limbi to investigate the cellular composition of the limbal niche and gene expression changes linked to innervation-dependent epithelial renewal. Their results revealed that Schwann cells, glial cells associated with tissue-innervating axon terminals, play a central role as regulators of corneal epithelial regeneration. Subsequent studies have identified additional biomarkers and biological processes involved in corneal epithelial wound healing. Beyond the previously reported biomarker *Creb5* discovered in a mouse model (Sun et al., 2024), Zhou et al. (2024) conducted scRNA-seq for the first time on corneal epithelial wound-healing samples derived from non-human primates (Cynomolgus monkeys). Their analysis highlighted the critical roles of limbal epithelial cells and basal epithelial cells in extracellular matrix formation and wound repair, as well as the importance of suprabasal epithelial cells in epithelial differentiation. Moreover, they identified *Thbs1* as a key biomarker of a transit-amplifying cell subcluster that promotes early stages of healing. Lu et al. (2025), from the same research group as Zhou, employed single-cell multiomics analysis, including scRNA-seq and single-cell assay for transposase-accessible chromatin using sequencing to characterize the early transcriptomic and chromatin accessibility changes of early wound response in mouse corneal epithelium, revealing a reduction in cell type-specific genes accompanied by an increase in common transcriptional responses, widespread alterations in chromatin accessibility across epithelial cell types, and a marked enrichment of *Fosl1/AP-1* binding sites within wound-induced open regions.

#### 3.1.2 Corneal stroma

**FIGURE 2:**
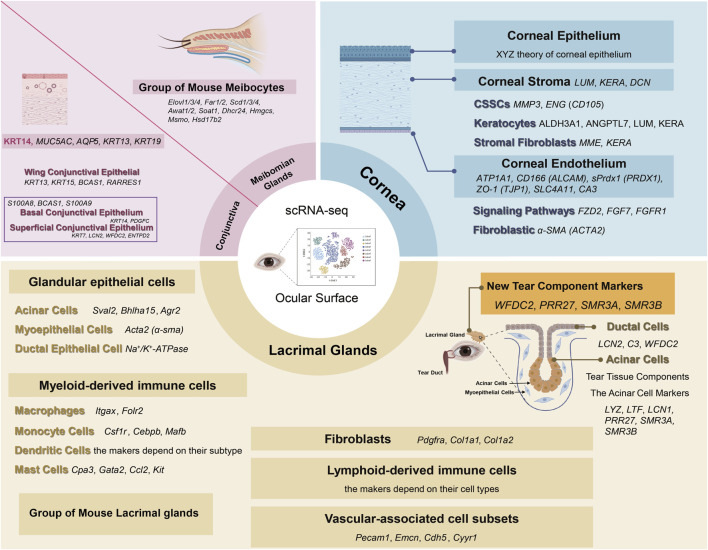
Application of scRNA-seq in healthy ocular surface structure and biomarkers. The cornea panel summarizes epithelium, stroma, CSSCs, keratocytes, stromal fibroblasts, and endothelium with representative biomarkers. The conjunctiva panel highlights epithelial, wing cells, and basal/superficial layers with representative biomarkers. The meibomian-gland panel depicts meibocytes enriched for lipid-synthesis genes. The lacrimal-gland panel shows glandular epithelium, fibroblasts, myeloid-derived immune cells, lymphoid-derived immune cells, vascular-associated cells, and tear-component with representative biomarkers. scRNA-seq, single-cell RNA sequencing; CSSC, corneal stromal stem cell. Created in BioRender. Wu, Y. (2025).

Comprising about 90% of the cornea’s thickness, the stroma is rich in collagen layers and populated mainly by keratocytes, which are key to preserving corneal biomechanics and transparency (Català et al., 2021; Ligocki et al., 2021; Hertsenberg and Funderburgh, 2015). Corneal stromal cells have been extensively examined in scRNA-seq studies. *LUM*, *KERA*, and *DCN* (Ligocki et al., 2021) are commonly used biomarkers for identifying human stromal cells. However, these studies have also identified biomarkers in corneal stromal cells that include stromal fibroblasts, keratocytes, and corneal stromal stem cells, with biomarkers varying across different studies. Collin et al. (2021) found *MMP3*, and *ENG* (*CD105*) as biomarkers for human corneal stromal stem cells. *ALDH3A1*, *LUM*, and *KERA* has also been discerned as a human keratocyte biomarker (Català et al., 2021). Additionally, van Zyl et al. (2022) used *MME* and *KERA* to pinpoint corneal stromal fibroblasts, and *ANGPTL7* to identify corneal stromal keratocytes (Figure 2). Human keratocyte function is also supported by high levels of matrix metalloproteinases *MMP2* and *MMP3*, metalloproteinase inhibitors *TIMP1* and *TIMP2*, adhesion molecules *THSB4*, *THSB1*, *ITGB4* and *ITGB1*, along with collagen-related biomarkers *COL12A1*, *COL6A3*, *COL1A2*, and *PCOLCE2* (Ligocki et al., 2021).

While scRNA-seq studies suggest heterogeneity within corneal stromal cells, it remains uncertain whether the identified populations represent distinct cell types or reflect varying activation states of keratocytes. For instance, biomarkers such as ENG are often used to define stromal stem cells, yet their expression is also associated with vascular cells (Mid et al., 2005). Similarly, elevated MMP and TIMP expression may indicate a dynamic response to tissue stress (Rudra et al., 2022).

#### 3.1.3 Corneal endothelium

Situated in the innermost layer of the cornea, the corneal endothelium is composed of a monolayer of hexagonal cells essential for maintaining corneal transparency (Chen et al., 2017). scRNA-seq of various human corneal tissues has revealed biomarkers for corneal endothelial cells, including *ATP1A1, CD166* (*ALCAM*), *sPrdx1* (*PRDX1*), *ZO-1* (*TJP1*), *SLC4A11*, and *CA3* (Català et al., 2021; Ligocki et al., 2021). Beyond these biomarkers, a cell expressing the myofibroblast biomarker *ACTA2* (*α-sma*) was identified as a fibroblastic corneal endothelial cell from human in Collin’s study (Collin et al., 2021). Additionally, genes involved in human corneal endothelial cell signaling pathways, such as *FZD2*, *FGF7*, and *FGFR1*, were identified (Ligocki et al., 2021) (Figure 2).

However, whether the observed transcriptional differences within corneal endothelial cells represent true biological subtypes or merely reflect variations in cell state, age, or experimental conditions remains unclear. For instance, the identification of ACTA2-expressing endothelial-like cells may suggest a population undergoing endothelial-to-mesenchymal transition (Wu et al., 2017), a process known to occur in disease states or during stress.

### 3.2 Conjunctiva

Several sequencing analyses of ocular tissues have now identified the presence of conjunctival cells. The utilization of a combination of biomarkers, notably *KRT14* (Collin et al., 2021; van Zyl et al., 2022), has proven instrumental in identifying conjunctival epithelial cells. Moreover, distinct biomarkers including *MUC5AC*, *AQP5*, *KRT13*, and *KRT19* have been applied for recognizing conjunctival epithelial cells (Li DQ. et al., 2021; Ligocki et al., 2021; Collin et al., 2021; van Zyl et al., 2022; Maiti et al., 2022; Gautam et al., 2021). In addition, biomarkers for basal conjunctival epithelium and superficial conjunctival epithelium, such as *S100A8*, *BCAS1*, and *S100A9*, were also identified. van Zyl et al. (2022) further used different biomarkers for basal (*KRT14, PDGFC*), superficial (*KRT7, LCN2, WFDC2, ENTPD2*), and wing (*KRT13, KRT15, BCAS1, RARRES1*) conjunctival epithelium (Figure 2).

However, it remains hard to distinguish true conjunctival epithelial subtypes from transitional or activated cell states based solely on scRNA-seq data. Several biomarkers, such as KRT19, are broadly expressed in stratified epithelial tissues (Natesan et al., 2019). Furthermore, biomarkers like S100A8 and S100A9 are known to be upregulated under other inflammatory conditions (Jukic et al., 2021), which may confound interpretations regarding basal or superficial epithelial identity. The identification of wing conjunctival epithelial cells based on WFDC2 expression also requires further validation, as these genes could be induced by environmental stress or microbial exposure (Ebihara et al., 2023).

### 3.3 Meibomian glands

Given that one of the principal functions of the meibomian glands is the secretion of lipids that contribute to the ocular surface tear film, Butovich and Wilkerson (2022) combined scRNA-seq with lipidomic and transcriptomic analyses for development and maturation of meibomian glands of C57Bl/6J founder mice to explore how gene expression and lipid composition change. In single-cell transcriptomic profiling of mouse tarsal plates, 14 cell clusters were identified in adult (about 80 days) samples, with 4 clusters (cluster IV, VI, IX, and XIII) showing high expression of meibogenesis-related genes, such as *Elovl1/3/4* (fatty acid elongation), *Far1/2* (fatty acyl reduction to fatty alcohols), *Scd1/3/4* (fatty acid desaturation), *Awat1/2* (acyl-CoA:wax alcohol acyltransferases producing wax esters), *Soat1* (cholesteryl ester synthesis), and cholesterol biosynthesis genes including *Hsd17b2*, *Msmo*, *Hmgcs*, and *Dhcr24*. These clusters were interpreted as meibocytes at different stages of differentiation or their progenitors. In the tarsal plates of pups (about 7 days), meibogenesis-rich cells were confined to a single cluster (cluster 16), with markedly fewer cells than in adults, reflecting the lower proportion of mature meibum lipids at this stage. Differential expression patterns were observed even within the same cluster, for example, *Far1* vs. *Far2* or *Scd3* vs. *Scd4*, suggesting subpopulation-specific specialization in lipid production.

However, at present, scRNA-seq studies on the meibomian glands remain limited, with no existing studies based on human tissue. This is likely because human meibomian glands have poor regenerative capacity, making living individuals unlikely to donate such low-regeneration tissues, and the number of cells obtainable from deceased individuals is extremely limited. Further research on healthy human samples may provide additional guidance for the management of meibomian gland-related diseases.

### 3.4 Lacrimal glands

scRNA-seq has been expanded to studies of the lacrimal gland, a critical organ responsible for tear production, ocular surface protection, lubrication, and maintaining surface homeostasis (Lin Y. et al., 2023). Tear secretion in the mature lacrimal gland is driven by the coordinated activity of acinar, ductal, and myoepithelial cells, all terminally differentiated and responsive to neural signals (Figure 2).

Several reports have studied the signaling mechanisms that control early lacrimal branching morphogenesis, revealing various mechanisms for the initial development of lacrimal glands (Dean et al., 2004; Ma et al., 2023; Hayashi et al., 2022). However, the exact developmental timeline of various cell types, their transcriptional characteristics, and progenitor cells in the lacrimal gland has yet to be determined. In 2017, Farmer et al. (2017) performed the first scRNA-seq of the lacrimal gland at two time points spanning key morphological changes during lacrimal gland development. They used scRNA-seq for the first time to reveal cellular composition, cellular dynamics, and lineage relationships in the developing lacrimal gland. Tracing different epithelial populations by lineage revealed novel features of epithelial homeostasis, providing the first direct evidence of a pool of progenitor cells in the lacrimal gland. Furthermore, Huang et al. (2024) and Fan et al. (2024) explored the heterogeneity and complexity (such as immune cell diversity) of murine extraorbital lacrimal gland through scRNA-seq and found over 37 subclasses of cells, including seven types of glandular epithelial cells (Acinar cells: co-expressing *Agr2*, *Bhlha15* and *Sval2*; Myoepithelial cells: co-expressing *Acta2* (*α-sma*); Ductal epithelial cells: co-expressing Na^+^/K^+^-ATPase), three types of fibroblasts (co-expressing *Col1a2*, *Col1a1* and *Pdgfra*), ten types of myeloid-derived immune cells (Macrophages: expressing *Itgax* or Folr2; Monocyte cells: two clusters co-express *Mafb*, *Cebpb* and *Csf1r*; Dendritic cells: the makers depend on their subtype; Mast cells: co-expressing *Kit*, *Ccl2*, *Gata2*, and *Cpa3*), at least eleven types of lymphoid-derived immune cells (the makers depend on their cell types), and five types of vascular-associated cell subsets (co-expressing *Cyyr1*, *Cdh5*, *Emcn*, and Pecam1) (Figure 2).

Recently, Bannier-Hélaouët et al. (2021) applied scRNA-seq technology to study human lacrimal gland tissues and organoids. They identified new tear component biomarkers *WFDC2*, PRR27, *SMR3A*, and *SMR3B* in lacrimal gland tissues. Through heterogeneity analysis of lacrimal gland acinar cells, various tear components were identified in addition to the expression of the acinar cell biomarkers *LYZ*, *LTF*, *LCN1*,* PRR27*,* SMR3A*, and *SMR3B*. Ductal cells predominantly expressed *LCN2*, *C3*, and *WFDC2*. Furthermore, they found that lacrimal gland organoid cells originated predominantly from ductal progenitor cells expressing *KRT*.

Despite the detailed cellular mapping provided by scRNA-seq studies of the lacrimal gland, several challenges remain in interpreting these findings. Many of the identified biomarkers, such as LCN2 and WFDC2, are not unique to the lacrimal gland and are expressed in other secretory tissues (Han et al., 2018). Additionally, the observed immune cell heterogeneity may be influenced by tissue processing or environmental stimuli rather than representing a stable resident population. It remains unclear whether the diverse immune cell subsets reflect steady-state physiology or potential inflammatory activation during sample preparation.

## 4 Application of single-cell RNA sequencing in the study of ocular surface diseases

Beyond its contributions to understanding physiological processes, scRNA-seq also plays critical role in investigating ocular surface disorders. Conditions like dry eye disease, keratoconus, Fuchs corneal endothelial dystrophy, corneal transplant rejection, and various other ocular surface diseases have been researched through this technology.

### 4.1 Dry eye disease

**FIGURE 3:**
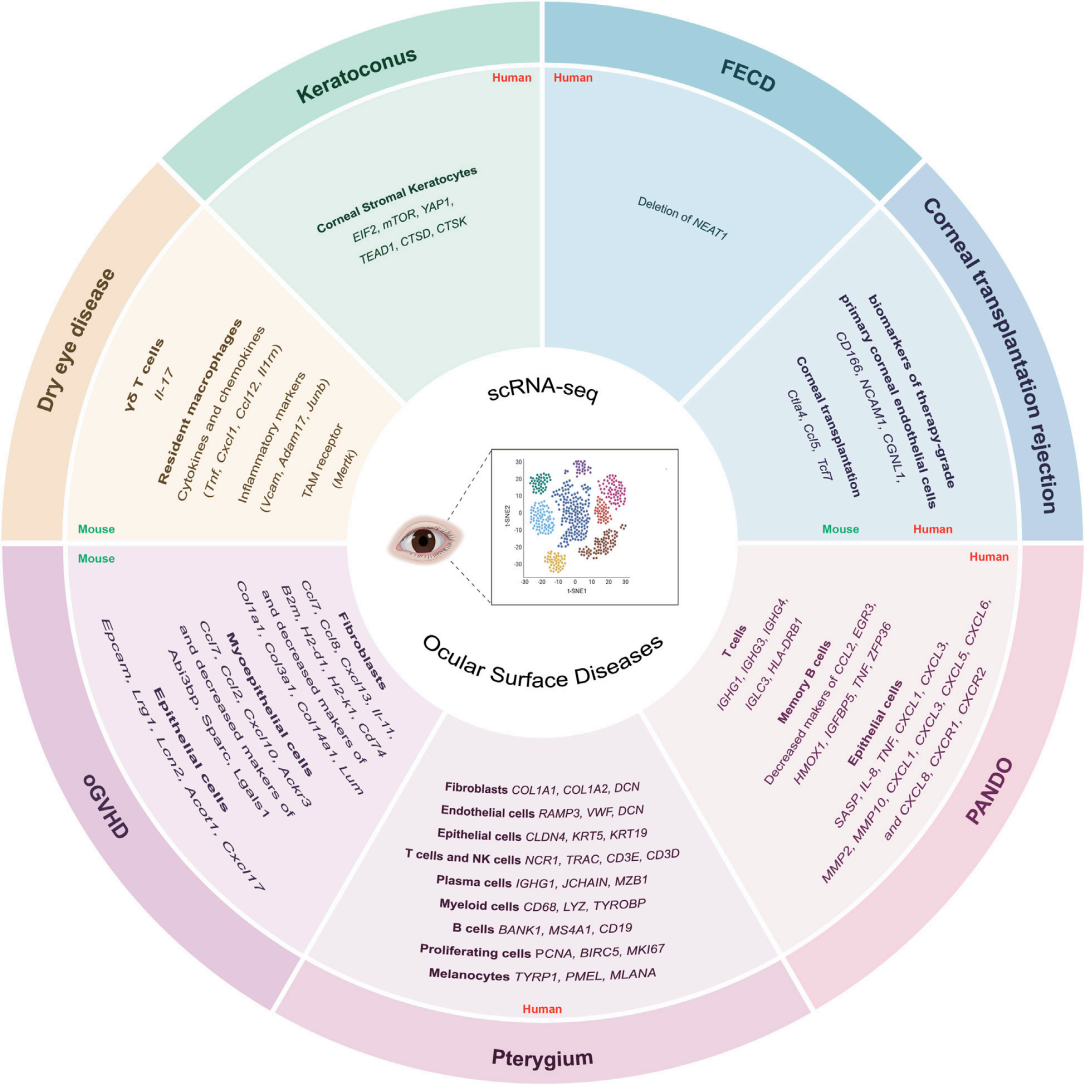
Application of scRNA-seq in ocular surface diseases and their biomarkers. These include dry eye disease, where immune and epithelial interactions are highlighted; pterygium, with altered epithelial, stromal, and immune cell populations; keratoconus, involving corneal stromal dysregulation; FECD, showing endothelial cell heterogeneity and regulatory pathways; and corneal transplantation rejection, marked by immune-mediated changes; Lacrimal gland-associated diseases, such as oGVHD and PANDO. FECD, Fuchs endothelial corneal dystrophy; oGVHD, ocular graft-versus-host disease; PANDO, primary acquired nasolacrimal duct. Created in BioRender. Wu, Y. (2025).

Dry eye disease, a multifactorial ocular surface disease, poses a significant threat to quality of life and may even lead to vision impairment or blindness (Craig et al., 2017; Kai et al., 2024; Qu et al., 2019). Numerous investigations have explored the pathogenesis of dry eye disease. Currently, tear film hyperosmolarity and ocular surface inflammation are considered the primary etiologies of dry eye disease (Hessen and Akpek, 2014; Yang et al., 2018; Lin S. et al., 2023), although comprehensive research remains essential. Recently, scRNA-seq tool has been used to the in-depth work within dry eye disease (Figure 3).

Alam et al. (2022) were the first to analyse the expression profile of conjunctival cells using scRNA-seq in C57BL/6J and RXRα mutant mice. Their investigation revealed distinct clusters of conjunctival immune cells in C57BL/6J mice. They compared gene expression in conjunctival immune cells, finding that γδ T cells were the main population expressing *Il-17*. Furthermore, they identified RXRα as an inhibitor of *Il-17* production by conjunctival lymphocytes, suggesting RXRα as a potential therapeutic target for dry eye disease. Following this, Alam et al. (2024) reported the scRNA-seq results of short-term dry eye disease-induced changes in corneal immune cell populations. The transition from monocytes to terminal resident macrophages was characterized, revealing significant differential expression across 1,365 genes. Resident macrophages exhibited increased expression of the *Tam* receptor (*Mertk*), inflammatory biomarkers (*Junb*, *Adam17*, and *Vcam*), and cytokines and chemokines (*Il1rn*, *Tnf*, Cxcl1, and *Ccl12*), along with decreased expression of complement and *MHCII* genes (Figure 3). Additionally, elevated levels of *Cxcl1* in the cornea intensified irritation and pain responses to topical hypertonic saline application. These findings suggest that these molecular changes contribute to the sensorineural alterations observed in dry eye disease. The similar scRNA-seq work by Liu et al. (2024) revealed a dry eye disease-associated pro-inflammatory microenvironment in the conjunctiva associated with dry eye disease. This microenvironment is centered around epithelial cells and involves interactions with macrophages and CD4 T cells, including Th1, Th17, and regulatory T cells (Treg).

In a recent publication (Lin JB. et al., 2023), scRNA-seq was employed to comprehensively profile the cellular landscape of the cornea under different physiological and pathological conditions, including healthy, dry eye disease, aging, and diabetic mouse models. This high-resolution analysis revealed five distinct cell populations that were specifically enriched or altered in the corneas of mice with dry eye disease, highlighting the cellular heterogeneity associated with the disorder. Notably, the study identified the matricellular protein SPARC as a key regulator during the adaptive regeneration of the corneal epithelium. SPARC was shown to modulate extracellular matrix remodeling and influence cell–matrix interactions, thereby facilitating epithelial cell migration and wound closure. Loss- and gain-of-function experiments further demonstrated that SPARC deficiency impaired epithelial repair, whereas its presence was indispensable for restoring barrier integrity.

### 4.2 Pterygium

Pterygium is a common, benign, wedge-shaped, fleshy tissue growth of the conjunctiva extending onto the cornea (Zhong et al., 2016; Zhang Y. et al., 2022). Zhang X. et al. (2024) utilized scRNA-seq to investigate the cellular transcriptional landscape of pterygium, revealing insights into its underlying pathogenesis and potential therapeutic targets. By comparing the conjunctival tissues of patients with primary pterygium to those of healthy individuals, significant differences in cell lineages were revealed. The study identified 9 major cell lineages in pterygium and normal conjunctival tissue: melanocytes (*MLANA*, *PMEL*, *TYRP1*), proliferating cells (*MKI67*, *BIRC5*, *PCNA*), B cells (*CD19*, *MS4A1*, *BANK1*), myeloid cells (*TYROBP*, *LYZ*, *CD68*), plasma cells (*MZB1*, *JCHAIN*, *IGHG1*), T cells and natural killer (NK) cells (*CD3D*, *CD3E*, *TRAC*, *NCR1*), epithelial cells (*KRT19*, *KRT5*, *CLDN4*), endothelial cells (*DCN*, *VWF*, *RAMP3*), and fibroblasts (*DCN*, *COL1A2*, *COL1A1*) (Figure 3). In addition, the study identified distinct sub-clusters of endothelial cells, epithelial cells, and fibroblasts. Various immune cell types were also analyzed, highlighting their potential roles in driving vascularization, inflammation, and immunosuppression in pterygium through dense cell-cell interactions. Notably, macrophages and ACKR1^+^ endothelial cells were found to potentially contribute to pterygium progression via intercellular communication. The findings further suggest that macrophages, recruited by ACKR1-activated vascular endothelial cells, may promote angiogenesis, immune suppression, and inflammatory responses, playing a pivotal role in pterygium development.

### 4.3 Keratoconus

Keratoconus is a progressive corneal disorder and the most prevalent form of primary ectasia, characterized by corneal thinning, irregular astigmatism, and reduced visual acuity (Dong et al., 2022). However, the factors contributing to the pathogenesis and progression of keratoconus are still not fully understood. The use of scRNA-seq technology offers a powerful tool to explore cellular heterogeneity in keratoconus.

In 2021, Collin et al. (2021) conducted the first scRNA-seq analysis of central corneal samples from individuals with keratoconus, comparing them to healthy central corneas. Their investigation revealed an increase in corneal stromal keratocytes in conical corneal samples, primarily driven by *EIF2* and *mTOR* signaling pathways, oxidative phosphorylation, and mitochondrial dysfunction. Recently, Dou et al. (2022) compared scRNA-seq analyses of central corneal cells from healthy individuals and those with keratoconus. They elucidated the cellular composition of the central cornea in both groups and confirmed the central role of stromal cells in the disease, highlighting the imbalance of collagen and extracellular matrix. They also identified potential new biomarkers of keratoconus, *YAP1*,* TEAD1*,* CTSD*, and *CTSK* (Figure 3).

### 4.4 Fuchs corneal endothelial dystrophy

The human corneal endothelium, a non-proliferative layer, typically remains quiescent. However, pathological conditions or specific ophthalmic procedures can damage the corneal endothelium, leading to endothelial loss and consequent visual impairment (Zhang et al., 2023). While single-cell transcription maps of endothelial cells across various tissues exist (Dumas et al., 2020; Kalucka et al., 2020), it remains uncertain whether corneal endothelial cells exhibit alterations in functional status at the single-cell level. Using scRNA-seq technology to reveal the heterogeneity of corneal endothelial cells at the single-cell level may provide insights into their functionality, pathological conditions, and the origins and mechanisms underlying corneal endothelial-related disorders (Yang et al., 2024).

Wang et al. (2022) performed the first high-resolution single-cell study of the human corneal endothelium. The sequencing results showed that the endothelium consists of heterogeneous cells with different transcriptional signals and functions. These endothelial cells were categorized into four clusters: C0-C3. These clusters were associated with oxygen level response and redox processes, translation elongation and peptide metabolism, cell cycle and DNA replication, and immune responses, respectively. Importantly, the study found that long noncoding RNA (lncRNA) NEAT1 was highly enriched in the C0 subpopulation but significantly downregulated in Fuchs endothelial corneal dystrophy (FECD). Further investigations confirmed the deletion of NEAT1 and its antioxidant role in the FECD model. Their findings suggest that NEAT1 may be a potential therapeutic target for FECD (Figure 3).

### 4.5 Corneal transplantation rejection

Corneal transplantation is a critical treatment modality for various ocular pathologies but faces limitations due to insufficient corneal donors and postoperative immune rejection (Li et al., 2024). Efforts to address these challenges have focused on developing alternative therapies that reduce reliance on donor corneas.

In 2015, Peh et al. (2015) pioneered a novel dual-media approach for propagating primary human corneal endothelial cells, making it possible to replace corneal transplantation with cell injection therapy. Subsequently, Kinoshita et al. (2018) successfully treated herpetiform keratitis by injecting human corneal endothelial primary cells supplemented with ROCK inhibitors, marking an important milestone in corneal endothelial cell injection therapy. However, the culture of corneal endothelial cells presents challenges. The culture process may lead to alterations in cell characteristics, and there is no standardized cellular benchmark for endothelial injections. Thus, understanding the heterogeneity of corneal endothelial cells across different generations is crucial. Català et al. (2023) recently performed single-cell sequencing of human primary corneal endothelial cells of different generations and culture times. This study revealed various cellular subtypes of human corneal endothelium generated from primary cultures and identified *CGNL1*, *NCAM1*, and *CD166* as biomarkers of therapy-grade primary corneal endothelial cells. These findings provide important references for cellular therapies for endothelial diseases of the cornea.

Additionally, rejection induced by allogeneic corneal transplantation remains a significant issue. Further studies on the mechanisms of this immune rejection are necessary. Lai et al. (2023) applied scRNA-seq towards corneas from mice transplanted with allogeneic corneas and found that the T-cell biomarker genes *Ctla4*, *Ccl5*, and *Tcf7* may play key roles in corneal transplant rejections, which may be achieved by affecting the activation of CD4^+^ T cells (Figure 3).

### 4.6 Lacrimal gland related diseases

#### 4.6.1 Ocular graft-versus-host disease

Ocular graft-versus-host disease (oGVHD) represents the most frequent complication following allogeneic hematopoietic stem cell transplantation (Bonelli et al., 2022). He et al. (2024) were the first to apply scRNA-seq to characterize lacrimal gland cell populations implicated in mouse oGVHD model, identifying 23 cell populations belonging to 11 cell types. They found that myoepithelial cells showed reduced secretion function, with lower expression of genes related to lipid metabolism and calcium channel activity. These cells also exhibited decreased extracellular matrix synthesis, indicated by downregulation of *Abi3bp, Sparc*, and *Lgals1*, and increased expression of chemokine-related genes *Ccl7*, *Ackr3*, *Ccl2*, and *Cxcl10*. Similarly, fibroblasts showed a reduction in extracellular matrix production, reflected by lower levels of *Lum, Col14a1, Col3a1*, and *Col1a1*. In contrast, they displayed an inflammatory gene expression pattern, characterized by elevated expression of cytokine and chemokine genes (*Ccl7*,* Ccl8*,* Cxcl13*,* Il11*) and increased levels of MHC class I-related genes (*H2-K1*,* H2-D1*,* B2m*) as well as the MHC class II-associated gene *Cd74*. Additionally, a newly identified epithelial population, referred to as Lrg1-high epithelial cells, characterized by increased levels of *Acot1*, *Lcn2, Cxcl17*, *Lrg1*, and *Epcam*, was found exclusively in the oGVHD lacrimal gland (Figure 3).

#### 4.6.2 Primary acquired nasolacrimal duct obstruction

Primary acquired nasolacrimal duct obstruction (PANDO) is a prevalent lacrimal drainage disorder in adults that severely impairs the lacrimal pump function responsible for transporting tears from the ocular surface to the nasal cavity (Ali, 2023). In scRNA-seq results of 5 PANDO patients lacrimal sac samples, Zhang W. et al. (2024) observed several key findings from 11 cell types were identified among 25,791 cells. They noted that T cells, which varied greatly in number, played an active role in the inflammatory response, showing higher expression of *HLA-DRB1*, *IGLC3*, *IGHG4*, *IGHG3*, and *IGHG1*. B cells were found to regulate the migration and proliferation of epithelial cells during the late inflammatory stage, with genes related to epithelial cell proliferation, such as *ZFP36*, *TNF*, *IGFBP5*, *HMOX1*, *EGR3*, and *CCL2*, being downregulated in memory B cells. Epithelial cells expressed *SASP* and upregulated inflammatory genes, including *MMP2, MMP10*, *CXCL3*, *CXCL1*, *TNF*, and *CXCL8 (IL-8)* during the late inflammatory stage. Additionally, neutrophils were recruited by epithelial cells through interactions between chemokines (*CXCL8*, *CXCL6*, *CXCL5*, *CXCL3*, and *CXCL1*) and their receptors (*CXCR2* and *CXCR1*), which were increased during the late inflammatory stage (Figure 3).

## 5 Summary and future perspectives

The integration of scRNA-seq technology has been used recently in a wide range of ocular tissues, including iris (Gautam et al., 2021), ciliary body (Youkilis and Bassnett, 2021), lens (Giannone et al., 2023), uvea (Zhang X. et al., 2022), choroid (Tong et al., 2024), retina (Mao et al., 2023), as well as cornea, conjunctiva, lacrimal gland, and lacrimal sac mentioned earlier. With the advancement of this technology, our understanding of the physiological mechanisms of ocular cells and the molecular pathology of ocular diseases will improve. This is due to the ability of scRNA-seq to identify not only key and typical biomarker genes of ocular cell types but also new and promising biomarker genes. Additionally, these studies have reported both common and rare cell states across different cell types and identified differences in gene expression among them.

Because the maintenance of ocular surface homeostasis is indispensable for clear vision, it is particularly important to characterize the normal functions of ocular surface cells and their alterations in disease at single-cell resolution. These cells are constantly exposed to the external environment and therefore highly susceptible to environmental insults, especially under extreme living or working conditions. Given this background, we comprehensively report the current application of scRNA-seq to the ocular surface, including the molecular identification of normal ocular surface cells and the molecular changes of these cells in disease states.

However, we have noted that similar scRNA-seq studies may present different insights about the same cells. This inconsistency may originate from various parts of the scRNA-seq procedures, including single-cell isolation and capture, cell lysis, reverse transcription, cDNA amplification, and sequencing library preparation. Another important source of variability among ocular surface scRNA-seq studies is the choice of sequencing platform. Although droplet-based methods such as 10x Genomics Chromium, Drop-seq, and inDrop share a similar overall workflow, their performances differ considerably in sensitivity, capture efficiency, and technical noise. Systematic benchmarking demonstrated that 10x Genomics offers the highest mRNA detection sensitivity and lower dropout rates, which is particularly advantageous when profiling rare or fragile cell types such as limbal stem cells or goblet cells, albeit at a higher per-cell cost (Zhang et al., 2019; Yamawaki et al., 2021). Drop-seq and inDrop provide more cost-effective solutions with scalable throughput, but suffer from reduced capture efficiency and increased barcode errors, potentially limiting their utility for small ocular tissues with low RNA content. Other platforms, such as ICELL8 or ddSEQ, can achieve high library efficiency or reduced multiplet rates, but generally detect fewer transcripts per cell compared to the latest 10x chemistries (Zhang et al., 2019; Yamawaki et al., 2021). For ocular surface applications, where sample quantity is often limiting and the preservation of delicate subpopulations is essential, the balance between sensitivity, throughput, and cost should be carefully considered when selecting a platform.

The importance of single-cell isolation and capture as the initial steps in scRNA-seq is self-evident. Therefore, we previously analysed the current main methods and proposed an optimized single-cell suspension for preparing mouse corneas based on previous attempts to ensure the high activity of cells (Liu et al., 2023). Arts et al. (2023) aimed to develop a comprehensive approach beginning with the final sequencing library, focusing on constructing a corneal meta-atlas by integrating all publicly available human corneal scRNA-seq datasets. This would provide a comprehensive biomarker gene and cell state reference, albeit at the cost of significant data storage and human maintenance. Furthermore, it is important to recognize that cell type identification in scRNA-seq studies inherently relies on clustering algorithms and the use of known biomarker genes, which may introduce classification biases (Chen et al., 2024). In many cases, the observed clusters might represent transitional cell states, stress-induced artifacts, or technical noise rather than distinct biological cell types (Livne and Efroni, 2024; Denisenko et al., 2020). The absence of universally specific biomarkers for certain populations further complicates accurate classification. Additionally, most studies infer cell identities solely based on transcriptomic similarity without further validation at the protein or functional level, increasing the risk of misinterpretation. This issue becomes particularly critical when analyzing rare cell types or disease-related subpopulations, where technical variation or batch effects may distort biological conclusions (Su et al., 2022). Another challenge limiting the use of scRNA-seq on the ocular surface is the relatively high cost. An entire scRNA-seq process could cost between $2,000 and $3,000 (Ali, 2023), depending on the supplier’s equipment and technical expertise, as well as the level of economic development in the location where it is performed. This financial barrier has significantly impacted the development of RNA-seq in the field of ocular surface research, reducing the number of related publications. Additionally, the complexity of data analysis requires researchers to invest substantial effort, highlighting the need for user-friendly analysis software.

Beyond the general challenges of scRNA-seq, several unique limitations arise when applying this technology specifically to ocular surface tissues. The ocular surface is anatomically thin, highly specialized, and composed of heterogeneous cell populations distributed across distinct regions such as the cornea, limbus, conjunctiva, and lacrimal glands. Due to the limited amount of biological material, particularly in small structures like the human limbus or conjunctiva, achieving sufficient cell numbers for robust single-cell analysis can be technically challenging. Additionally, the isolation of ocular surface cells generally requires enzymatic dissociation, for instance, the optimized corneal protocol (Liu et al., 2023), or alternative approaches such as magnetic bead-activated cell sorting and post-sort culture optimization (Vattulainen et al., 2024). Nevertheless, these methods may still perturb cellular states or activate stress-responsive genes, thereby complicating downstream transcriptomic analyses. Some fragile cell types, including goblet cells or rare limbal progenitors, are especially prone to loss during dissociation or under-representation in sequencing outputs. Another concern is that most of the newly proposed biomarkers, for example, SOX17^+^/TSPAN7^+^ for LSCs, are supported solely by transcriptomic evidence and lack validation through *in vitro* assays. In addition, recently identified disease-associated biomarkers (CTSD and CTSK) may have limited relevance for clinical diagnosis, as only one publication has reported them as biomarkers for keratoconus, and no follow-up studies have been published in the past 3 years; therefore, further comparative analyses against established clinical indicators are warranted. Furthermore, the ocular surface exhibits pronounced spatial heterogeneity-cells of the central cornea, peripheral limbus, and conjunctiva perform distinct functions but may express overlapping biomarkers, complicating cell identity assignment when spatial context is lost. This spatial limitation is further compounded by the absence of positional information in most scRNA-seq datasets, which hinders the ability to distinguish true cell type differences from regional expression variability.

Future integration of spatial transcriptomics, multi-omics approaches, and advanced computational deconvolution methods may help address these issues (Du J. et al., 2023), enabling more precise characterization of ocular surface cell types and states. Spatial transcriptomics complements scRNA-seq by preserving the positional context of cells within intact tissue, thereby enabling the distinction between transcriptional variation driven by intrinsic cell-type heterogeneity and that shaped by the surrounding microenvironment (Longo et al., 2021). In ocular surface research, this is particularly critical for distinguishing limbal stem cells from morphologically similar epithelial progenitors and for mapping immune cell infiltration patterns in disease states. Furthermore, multi-omics approaches, which integrate genomic, transcriptomic, epigenomic, proteomic, and metabolomic profiles at the single-cell level, offer a holistic view of cellular identity and function (Fan et al., 2025). Such integration can validate transcriptome-derived cell-type classifications by linking them to protein expression or metabolic activity, thereby reducing misclassification caused by reliance on RNA biomarkers alone.

In addition to these integrative approaches, a major technical confounder in ocular surface scRNA-seq might be dissociation-induced stress, which rapidly triggers immediate-early response genes (such as Fos, Jun, Arc) (Denisenko et al., 2020; Machado et al., 2021; Lacar et al., 2016), heat-shock proteins (Denisenko et al., 2020; Neuschulz et al., 2023), and inflammatory cytokines (O'Flanagan et al., 2019), potentially masking native transcriptional states. Mitigation strategies include minimizing enzymatic digestion time, performing low-temperature dissociation with cold-active proteases, applying *in situ* fixation or transcriptional inhibitors, and optimizing mechanical handling to reduce cellular activation (Machado et al., 2021). One effective approach to circumvent dissociation-induced artifacts is to bypass whole-cell isolation altogether. For delicate and low-yield ocular tissues, single-nucleus RNA-seq can avoid enzymatic and mechanical dissociation and better preserve native signatures (Machado et al., 2021); however, it has trade-offs such as lower transcript capture, loss of cytoplasmic RNAs, and underrepresentation of certain cell types (Bakken et al., 2018). The choice between scRNA-seq and snRNA-seq should be guided by tissue fragility, target cell abundance, and the resolution required for the research question.

When these challenges are alleviated by future technologies, it will be possible to report in detail on more common ocular surface diseases affecting visual acuity that are currently underreported. Examples include various ocular surface diseases, such as corneal neovascularization after surgery or associated simultaneously with other ocular surface diseases, and meibomian gland dysfunction affecting ocular surface comfort and tear film stability (Wang et al., 2016). In addition, the growing repertoire of validated cell-type-specific and disease-associated biomarkers identified through scRNA-seq might hold substantial clinical translational potential, for example, the ACKR1+ endothelial cells in pterygium (Zhang X. et al., 2024) could point toward novel therapeutic targets. Such biomarkers could facilitate early diagnosis of ocular surface disorders, especially as short-term symptoms may lead to transcriptome changes in dry eye disease (Alam et al., 2024), enable more precise patient stratification, and inform targeted therapeutic strategies, including cell-based regenerative approaches and molecular interventions aimed at restoring ocular surface function.

## 6 Method of literature search

A comprehensive search was performed in the PubMed and Web of Science databases (up to September 2024), without limitations on publication date or type. Articles not published in English or lacking peer review were excluded. This narrative review incorporated a range of relevant keywords and phrases, including but not limited to: “single cell sequencing,” “single-cell sequencing,” “ocular surface,” “cornea,” “conjunctiva,” “meibomian glands,” “lacrimal gland,” “keratoconus,” and “pterygium.”
